# Differentially-Expressed miRNAs in Ectopic Stromal Cells Contribute to Endometriosis Development: The Plausible Role of miR-139-5p and miR-375

**DOI:** 10.3390/ijms19123789

**Published:** 2018-11-28

**Authors:** Kadri Rekker, Tõnis Tasa, Merli Saare, Külli Samuel, Ülle Kadastik, Helle Karro, Martin Götte, Andres Salumets, Maire Peters

**Affiliations:** 1 Department of Obstetrics and Gynecology, Institute of Clinical Medicine, University of Tartu, 50090 Tartu, Estonia; merli.saare@ut.ee (M.S.); helle.karro@kliinikum.ee (H.K.); andres.salumets@ccht.ee (A.S.); maire.peters@ut.ee (M.P.); 2 Competence Centre on Health Technologies, 50410 Tartu, Estonia; kylli.samuel@ccht.ee; 3 Institute of Computer Science, University of Tartu, 50090 Tartu, Estonia; ttasa@ut.ee; 4 Tartu University Hospital’s Women’s Clinic, 50406 Tartu, Estonia; ylle.kadastik@kliinikum.ee; 5 Department of Gynecology and Obstetrics, University of Münster, 48149 Münster, Germany; martin.goette@ukmuenster.de; 6 Department of Biomedicine, Institute of Biomedicine and Translational Medicine, University of Tartu, 50090 Tartu, Estonia; 7 Department of Obstetrics and Gynecology, University of Helsinki and Helsinki University Hospital, FI-00014 Helsinki, Finland

**Keywords:** endometriosis, ectopic stroma, microRNA, small RNA sequencing, *EDN1*, *HOXA10*, miR-139-5p, miR-375

## Abstract

microRNA (miRNA) expression level alterations between endometrial tissue and endometriotic lesions indicate their involvement in endometriosis pathogenesis. However, as both endometrium and endometriotic lesions consist of different cell types in various proportions, it is not clear which cells contribute to variability in miRNA levels and the overall knowledge about cell-type specific miRNA expression in ectopic cells is scarce. Therefore, we utilized fluorescence-activated cell sorting to isolate endometrial stromal cells from paired endometrial and endometrioma biopsies and combined it with high-throughput sequencing to determine miRNA alterations in endometriotic stroma. The analysis revealed 149 abnormally expressed miRNAs in endometriotic lesions, including extensive upregulation of miR-139-5p and downregulation of miR-375 compared to eutopic cells. miRNA transfection experiments in the endometrial stromal cell line ST-T1b showed that the overexpression of miR-139-5p resulted in the downregulation of homeobox A9 (*HOXA9*) and *HOXA10* expression, whereas the endothelin 1 (*EDN1*) gene was regulated by miR-375. The results of this study provide further insights into the complex molecular mechanisms involved in endometriosis pathogenesis and demonstrate the necessity for cell-type-specific analysis of ectopic tissues to understand the interactions between different cell populations in disease onset and progression.

## 1. Introduction

Endometriosis is a fibrotic condition defined by the presence and growth of endometrial-like tissue outside the uterine cavity [1]. Current endometriosis treatment strategies are rather general and mainly alleviate pain symptoms, but there are no specific approaches to cure the disease [2]. Therefore, the elucidation of aberrant molecular processes in endometriotic tissues is necessary to find new molecules enabling targeted endometriosis therapies. Various molecular aberrations between endometriotic lesions and eutopic endometrium have already been detected, which can partly explain the disease pathogenesis (reviewed in [3,4]); nevertheless, the complete molecular etiology of endometriosis is still unclear. 

microRNAs (miRNAs) are non-coding RNA molecules of ~22 nucleotides in length that have a regulatory function in gene expression. One miRNA can regulate more than 100 genes [5] and, in turn, a single gene can be regulated by multiple miRNAs [6]. miRNAs regulate the translation of target mRNAs negatively; however, through a combinatorial action of miRNAs and transcription factors (TFs), more complex regulatory networks are often involved in various biological events [7]. Accumulating evidence also indicates the involvement of miRNAs in the development and persistence of endometriosis [8,9,10,11,12]. Nevertheless, there is a lack of consistency among reported lists of aberrantly-expressed miRNAs, with the most likely underlying cause being the variation in the cellular composition of the studied tissue [13]. To overcome the issue of study material heterogeneity, Logan et al. investigated mRNA and miRNA expression in pure fractions of uncultured eutopic endometrial epithelial and stromal cells of endometriosis patients compared to control women and found that differentially-expressed miRNAs in stromal cells were distinct from miRNAs in epithelial cells [14]. Recently, we demonstrated that the investigation of uncultured eutopic and ectopic stromal cells of endometriosis patients unveils transcriptomic differences that may remain unnoticed in whole-tissue examination, stressing the importance of cell-type-specific analysis [15]. However, there are no high-throughput studies revealing miRNA expression patterns from distinct cell types in ectopic tissues.

In the current study, we applied small-RNA sequencing to uncultured paired eutopic and ectopic endometrial stromal cells to reveal disease-specific alterations. To understand the regulatory networks between genes and miRNAs, we implemented an integrated analysis of mRNA data from our previous study [15] and differentially-expressed miRNAs from this study.

## 2. Results

### 2.1. miRNA Profile of Eutopic and Ectopic Endometrium

miRNA expression profiles from uncultured endometrial stromal cells from paired samples of eutopic endometrium (*n* = 4) and endometriomas (*n* = 4) were determined by small RNA sequencing. In total, 719 miRNAs were detected in eutopic and 637 miRNAs in ectopic stroma (present in at least 50% of samples, Table S1). Most abundant miRNAs were highly similar in both groups (Table 1), where let-7a-5p was the most highly-expressed miRNA in stromal cells of eutopic and ectopic origin.

High-throughput sequencing revealed 149 differentially-expressed miRNAs (recognized by at least 2/3 analysis methods, adjusted *p*-value < 0.05, log_2_ fold change (|log_2_FC| ≥ 1)), where 71 miRNAs were downregulated and 78 miRNAs upregulated in ectopic stromal cells (Table S2, Figure S1). Twenty-one miRNAs were recognized as differentially-expressed between eutopic and ectopic endometria with all three methods used (Table 2). miR-139-5p was statistically most significantly upregulated [log_2_FC = 5.0, false discovery rate (FDR) = 1.4 × 10^−24^] and miR-375 most significantly downregulated (log_2_FC = −4.9, FDR = 1.4 × 10^−14^ in ectopic stroma.

Eight potential novel miRNAs were determined from small RNA sequencing data of eutopic and ectopic stromal cells (Table 3). One candidate miRNA (provisional ID: 3_18752) was detected only in eutopic stromal cells. All other sequences were detected from both eutopic and ectopic stroma; however, none of the novel miRNAs were differentially expressed. Three potential novel miRNAs showed similarities with other human miRNAs according to the miRBase database.

### 2.2. miRNA Validation by Quantitative Real-Time PCR (qRT-PCR)

**Figure 1:**
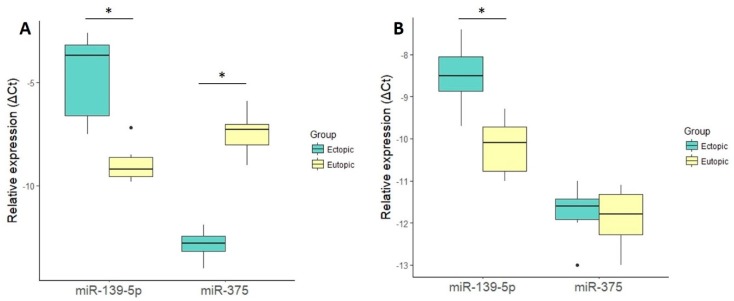
Relative miRNA expression levels (log2 scale) in (**A**) paired uncultured eutopic (*n* = 6) and ectopic (*n* = 6) stromal cells and (**B**) paired cultured eutopic (*n* = 6) and ectopic (*n* = 6) stromal cells. The ΔCt values were calculated as follows: miRNA Ct value − average *C*t value of reference genes (RNU44 and RNU48). * *p*-value < 0.05. Outliers (defined as datapoints outside 1.5 times the interquartile range above the upper quartile and below the lower quartile) are pointed out with black dots. For illustrative purposes, relative expression levels (Δ*C*t) were multiplied by −1.

Small RNA-sequencing data validation from six pairs of fluorescence-activated cell sorting (FACS)-isolated eutopic and ectopic stromal cells by qRT-PCR confirmed the upregulation of miR-139-5p (FC = 19, *p* = 0.03) and downregulation of miR-375 (FC = −42, *p* = 0.03) in ectopic stroma (Figure 1A). To verify whether the detected miRNA alterations could also be identified in cultured cells, miR-139-5p and miR-375 levels were determined in six paired cultured eutopic and ectopic stromal cells. A slight upregulation of miR-139-5p (FC = 3.2, *p* = 0.03) in cultured ectopic cells was determined, but no differential expression was detected for miR-375 (> 0.05, Figure 1B), indicating the effect of cell culturing on miRNA expression levels.

### 2.3. Integrated miRNA–mRNA Analysis for Target Identification

Target gene prediction was performed using MAGIA^2^ (http://gencomp.bio.unipd.it/magia2), which identifies two types of regulatory circuits: (a) TFs that regulate both miRNAs and their targets; and (b) miRNAs that regulate both TFs and their targets. In the current study, MAGIA^2^ identified 5914 significant links (*q*-value < 0.05) between TFs and their targets (miRNAs or mRNAs) and 1183 significant links between miRNAs and their targets (miRNA/mRNA or miRNA/TF, Table S3). The top 200 interactions are visualized in Figure S2. Among others, several interesting TFs including estrogen receptor 1 (*ESR1*), signal transducer and activator of transcription (STAT) genes (*STAT2*, *STAT3*, *STAT5A*, *STAT5B*), v-rel avian reticuloendotheliosis viral oncogene homolog A (*RELA*) and nuclear factor kappa B subunit 1 (*NFKB1*) were predicted to regulate miRNAs that were determined to be differentially-expressed in our study. In addition, various mRNAs such as mitogen-activated protein (MAP) kinases and insulin like growth factor 1 (*IGF1*) were determined as targets for differentially-expressed miRNAs (Table S3).

As no targets for miR-139-5p and miR-375 were predicted by the MAGIA^2^ program, an additional target prediction analysis for these miRNAs was conducted using the DIANA microT (https://bio.tools/DIANA-microT), TargetScan (http://www.targetscan.org), miRanda (https://omictools.com/miranda-tool) and miRDB (http://www.mirdb.org) programs. Target genes predicted by at least two programs and that showed differentially-expressed levels (downregulated for miR-139-5p and upregulated for miR-375) in our previous study [15] were considered as potential targets for miR-139-5p and miR-375. For miR-139-5p, 16 potential targets were found, and for miR-375, 19 potential targets were found (Table S4).

### 2.4. miRNA Target Validation

To examine the impact of miR-139-5p and miR-375 on target gene expression in stromal cells, the cell line ST-T1b was transfected with selected miRNA precursors or negative control precursors and the expression of seven and 10 potential target genes for miR-139-5p and miR-375, respectively, were selected for validation by qRT-PCR (Table S4). The overexpression of miR-139-5p resulted in 2.1-fold and 1.8-fold downregulation of *HOXA9* (*p* = 0.0005) and *HOXA10* (*p* = 0.001) expression, respectively. The overexpression of miR-375 resulted in a 1.9-fold downregulation of *EDN1* gene expression (*p* = 0.01). The expression of the other tested target genes (*CDH20*, *ESRRG*, *FBN2*, *LRFN5, GNAO1* for miR-139-5p and *ZFPM2, GATA6, FZD4, AHR, CD200, CTGF, DUSP6, IL1RAP, NCAM1* for miR-375) did not differ between the transfected cells (all *p* ˃ 0.05).

## 3. Discussion

To the best of our knowledge, this is the first study utilizing an uncultured cell-type specific approach and high-throughput small-RNA sequencing for miRNA analysis of endometriotic lesions. We demonstrated distinct alterations in miRNA expression patterns between uncultured stromal cells from the endometrium and endometriomas and their potential involvement in miRNA-mediated pathological processes occurring in endometrial cells in ectopic locations.

According to our analysis, the statistically most significantly upregulated miRNA in ectopic stromal cells was miR-139-5p. The involvement of this miRNA in endometriosis had previously not been determined; however, decreased expression of miR-139-5p occurred in endometrial cancer tissues [16] where its levels are inversely correlated with *HOXA10* expression [17]. The suppressive impact of miR-139-5p on *HOXA10* gene expression was confirmed in our study, and in addition we observed a downregulation of another homeobox gene, *HOXA9*. A lower level of *HOXA10* expression in ovarian endometriomas and peritoneal endometriotic lesions in contrast to eutopic endometrium has been previously detected and it has been speculated that aberrant *HOXA10* expression might contribute to endometriosis pathogenesis through progesterone resistance [18] or by the induction of autophagy [19]. Both HOX genes are also highly expressed in the endometrium and play important roles in endometrial receptivity [20]. *HOXA10* expression is lower in the mid-secretory endometrium during the implantation window in endometriosis patients [21], but the levels are restored after surgical resection of endometriotic tissue [22]. Therefore, aberrant HOX gene levels likely contribute to the etiology of infertility in patients with endometriosis. 

Furthermore, an integrated analysis of miRNA–mRNA expression data by MAGIA^2^ predicted that miR-139-5p is regulated via estrogen receptor alpha (encoded by *ESR1* gene; Table S3). Although there are contradicting results regarding *ESR1* expression in ectopic endometrium, in particular ovarian endometriosis [23,24,25], it is widely accepted that the *ESR1* gene has a pivotal role in endometriosis pathogenesis. *HOXA10* expression is also regulated by estrogen receptor alpha [26], suggesting sophisticated molecular interactions between miRNAs, their targets and transcription factors. 

The most significantly downregulated miRNA in our dataset was miR-375. In contrast to miR-139-5p, the downregulation of miR-375 was only observed in FACS-isolated cells, but got lost upon in vitro culture, emphasizing the importance of performing investigations in uncultured cells. The downregulation of miR-375 has been consistently reported in previous endometriosis studies involving whole eutopic and ectopic tissues [8,9,10,12]; however, the possible function of miR-375 in endometriosis has not been elucidated. We found that one of the miR-375 predicted targets was the *EDN1* gene, which is expressed more highly in stromal cells from ectopic origin compared to eutopic endometrium [15], and confirmed the potential regulatory link between the miR-375 and *EDN1* gene by transfection experiments. However, a comprehensive review has revealed that besides tissue-specific miRNA-mediated regulation, *EDN1* gene transcription may be modulated by DNA methylation and histone modification patterns, as well as being influenced by different transcription factors responding to a wide variety of stimuli [27]. Therefore, the impact of miR-375 overexpression, detected in in vitro conditions, is probably less straightforward in in vivo situations. In order to determine whether the downregulation of miR-375 results in higher levels of *EDN1* in ectopic stroma, experiments with anti-miR-375 should be performed. However, as the baseline level of miR-375 in ST-T1b endometrial stromal cell line is low, it is unlikely that the further repression of miR-375 with antagomiR could show a considerable effect on *EDN1* gene levels. 

Nevertheless, endothelin-1 (ET-1), which is encoded by the *EDN1* gene, has been associated with endometriosis pathogenesis, as the cystic fluid of endometriomas contains a higher amount of ET-1 compared to ovarian cysts other than endometriomas, and in vivo experiments in mice demonstrated that blocking ET-1 activity was effective in decreasing endometriosis-related pain [28]. Also, ET-1 supports the survival, angiogenesis and migration of mesenchymal stem cells [29], which are also proposed to be involved in endometriosis development [30,31]. Thus, the suppression of *EDN1* transcription by the overexpression of miR-375 could potentially be used as a therapy for endometriosis-related pain or as a strategy to prevent the dissemination of endometrial mesenchymal stem cells outside the uterus.

Besides miR-375, we detected several other dysregulated miRNAs that have been previously reported as being differentially-expressed in endometriosis studies investigating whole eutopic and ectopic tissues. Although the overlap between previously published miRNA studies has remained minimal, constant downregulation of miR-200-family members (miR-200a, miR-200b, miR-200c and miR-141), miR-196b-5p, miR-183-3p, miR-34c-5p, and upregulation of miR-202 in ectopic compared to eutopic tissue has been reported [13]. As our study confirmed the differential expression of these miRNAs in endometriotic stromal cells, we suggest that the aforementioned miRNAs most likely contribute to endometriosis pathogenesis and/or the persistence of the disease. 

In conclusion, our cell-type-specific analysis revealed remarkable differences in miRNA expression patterns between stromal cells isolated from the endometrium and endometriomas. Based on our findings, we propose that two molecular mechanisms are involved in endometriosis pathogenesis, where, firstly, *HOXA9* and *HOXA10* genes are regulated by miR-139-5p among other factors and are potentially involved in endometriosis-associated infertility. Secondly, the aberrant expression of miR-375 in ectopic stromal cells may contribute to higher levels of *EDN1* in lesions, which can be associated with pain mechanisms or be involved in the regulation of invasive growth and cell proliferation in endometriosis development. Further functional studies are still needed to prove the connections between these miRNAs and endometriosis development. Nevertheless, the current results provide evidence that further studies are needed to learn about the interactions within and between all cell populations of endometriotic lesions and to uncover the exact molecular mechanisms behind the disease pathogenesis.

## 4. Materials and Methods 

### 4.1. Patients and Sample Collection

The study was approved by the Research Ethics Committee of the University of Tartu, Estonia (approval no. 278/M-18; approval date: 19 February 2018). Patients undergoing laparoscopic surgery at Tartu University Hospital Women’s Clinic with symptoms of endometriosis were recruited and signed informed consent was obtained from all women who entered the study. In total, 12 patients aged 32.0 ± 6.6 years (mean ± standard deviation) and with a body mass index of 22.4 ± 2.4 kg/m^2^ were enrolled. According to the revised American Society for Reproductive Medicine classification system [32], the severity of the disease was classified as moderate–severe (stage III–IV) in all cases. None of the participants had received hormonal treatments for at least three months prior to the time of sample collection. 

Biopsies from endometriomas and eutopic endometria were obtained at the proliferative menstrual cycle phase and were processed and preserved as described previously [15]. Briefly, the collected tissue samples were subdivided, immediately immersed into formalin for histopathological assessment or into the cryopreservation medium, cooled down at −80 °C freezer overnight and subsequently kept in liquid nitrogen until further use. Histopathological evaluation was performed on endometrioma samples and the diagnosis of endometriosis was confirmed in all cases. 

### 4.2. Stromal Cell Isolation from Eutopic and Ectopic Endometria

Stromal cells were isolated from paired endometrial (*n* = 6) and endometrioma (*n* = 6) biopsies using fluorescence-activated cell sorting (FACS) as described previously [15]. Cells were stained with phycoerythrin-conjugated mouse anti-human CD10 antibody (1:20 dilution, clone HI10a, BD Pharmingen, San Diego, CA, USA) and were sorted into 1× PBS (without Ca^2+^ and Mg^2+^). Total RNA was isolated immediately using miRNeasy Micro kit (Qiagen, Hilden, Germany). The quality and quantity of isolated RNA was assessed with 2200 TapeStation RNA ScreenTape (Agilent Technologies, Palo Alto, CA, USA).

### 4.3. Small RNA Sequencing

Endometrial stromal cells from paired samples of eutopic endometrium (*n* = 4) and endometriomas (*n* = 4) isolated by FACS were subjected to high-throughput small RNA sequencing. Library construction and sequencing were performed by an external service provider (Admera Health LLC, South Plainfield, NJ, USA). Small RNA libraries were prepared using NEBNext Small RNA Library Prep kit (New England Biolabs, Ipswich, MA, USA) and sequencing was performed with 1 × 76 bp NextSeq High Output kit on NextSeq 500 platform (Illumina, Inc, San Diego, CA, USA).

### 4.4. Sequencing Data Analysis

Small RNA sequencing data were deposited into the Gene Expression Omnibus (GEO accession number GSE121406). The quality of the input reads before and after the read trimming was assessed with FastQC v0.9.5 (https://www.bioinformatics.babraham.ac.uk/projects/fastqc/). Read trimming and filtering was performed with Cutadapt v1.8.1 (https://cutadapt.readthedocs.io/en/stable/) [33]. All reads shorter than 15 and longer than 35 base-pairs, adapter sequence and sequence read ends with quality value less than 15 were filtered out. The read-mapping tool STAR aligner v2.4.0j (http://code.google.com/p/rna-star/) [34] was used to align quality-controlled sequences to the human reference genome (GRCh38). miRNA alignment filtered out reads with (a) fewer than 17 bp matched to reference (--outFilterMatchNmin), (b) alignments matched to more than five locations (--outFilterMultimapNmax) and (c) if more than 5% of the total read length was mismatched (--outFilterMismatchNoverLmax). No separate restrictions were set on the number of matched bases relative to the read length or alignment score. Raw counts of the miRNA regions were quantified using featureCounts v1.5.2 (bioinf.wehi.edu.au/featureCounts/) that allowed strand-specific and multiply-aligned reads. Successfully-aligned reads were then quantified against mature *Homo sapiens* miRNA coordinates retrieved from miRBase version 21 (http://www.mirbase.org/) [35]. Novel miRNA sequences were predicted with miRDeep2 (https://www.mdc-berlin.de/n-rajewsky#t-data, software&resources) [36] using default settings. Reads predicted as potential candidate miRNAs by miRDeep2 were subjected to BLAST to discriminate the sequences corresponding to other human coding or non-coding RNAs. Sequences were considered as candidate novel miRNAs if detected in at least two out of eight sequenced samples.

Differentially-expressed miRNAs between stromal cells from eutopic and ectopic endometria were identified using edgeR v3.16.5 [37], DESeq2 v1.14.1 [38] and baySeq v2.8.0 [39] packages. Final p-values were reported as corrected for multiple testing with FDR for edgeR and baySeq and the Benjamini–Hochberg method for DESeq2. miRNAs were considered as differentially-expressed if recognized by at least two out of three methods (adjusted *p*-values ≤ 0.05). A heatmap was generated with ClustVis (https://biit.cs.ut.ee/clustvis/) [40].

### 4.5. miRNA Target Prediction

An integrated analysis of differentially-expressed miRNAs combined with mRNAs from paired eutopic and ectopic endometrial stromal cells from our previous study [15] was used to predict miRNA targets. The software MAGIA^2^ [41] was implemented to construct post-transcriptional regulatory networks including circuit components of TF regulating both miRNA and its target mRNA, and miRNA regulating both TF and its target. Raw counts for both miRNA and mRNA samples were normalized using weighted trimmed mean of M-values (TMM) normalization implemented in edgeR. A combined meta-analysis was applied as the samples used for mRNA and miRNA analysis originated from different women. Three different target prediction methods within MAGIA^2^ were used with mean stringency: RNA22 (threshold: −27.4) [42], DIANA microT (threshold: 2.7) [43] and TargetScan (threshold: 0.7) [44]. 

### 4.6. miRNA Transfection

Transfection experiments were performed for the validation of the predicted miRNA targets. The immortalized endometrial stromal cell line ST-T1b [45] was cultured in medium containing 70% Dulbecco’s Modified Eagle’s medium (PAA Laboratories, Pasching, Austria), 18% MCDB-105, 10% fetal bovine serum, 1% glutamine, 1% penicillin/streptomycin and 5 µg/ml insulin. For miRNA transfection, cells were plated in six-well plates one day before transfection to reach 70% confluency. Cells were then transfected with the miRNA precursors miR-139-5p, miR-375 or pre-miR precursor negative control #2 (Thermo Fisher Scientific, Waltham, MA, USA), via lipotransfection with DharmaFECT reagent (Thermo Fisher Scientific, Waltham, MA, USA) in OPTI-MEM media (Life Technologies, Grand Island, NY, USA) according to the manufacturer’s instructions. Twenty-four hours later, the medium was replaced by respective normal culture medium. Expression analyses of predicted miRNA targets for miR-139-5p and miR-375 (Table S4) were performed 48 to 72 h after transfection. To confirm the efficiency of miRNA transfection, miR-139-5p and miR-375 levels were determined by qRT-PCR from ST-T1b stromal cells transfected with miR-139-5p, miR-375 or precursor negative control. The experiments were conducted as three separate transfections resulting in a total of eight replicate samples for both miRNAs and eight control samples. RNA was isolated from the cells using innuPREP RNA Mini Kit (Analytik Jena AG, Jena, Germany) following the manufacturer’s instructions. 

### 4.7. Primary Cultures of Human Endometrial Stromal Cells

Primary cultures of human endometrial stromal cells were prepared from paired endometrial (*n* = 6) and endometrioma (*n* = 6) biopsies. Single cell suspensions of endometrial stromal cells for primary culture were isolated and cultured as described previously [46]. Confluent cells were collected and stored in RNAlater (Thermo Fisher Scientific, Waltham, MA, USA) at −80 °C until further analysis.

### 4.8. qRT-PCR

miR-139-5p (Applied Biosystems, Assay ID 005364) and miR-375 (ID 000564) expression levels from uncultured and cultured stromal cells were validated by qRT-PCR. cDNA synthesis was conducted with TaqMan MicroRNA Reverse Transcription Kit (Thermo Fisher Scientific, Waltham, MA, USA) and qRT-PCR was performed with TaqMan Universal PCR Master Mix, No AmpErase UNG (Thermo Fisher Scientific, Waltham, MA, USA). RNU44 (ID 001094) and RNU48 (ID 001006) were used as references for normalization. Real time PCR experiments were performed in duplicate using 7500 Fast or ABI PRISM 7300 Real Time PCR Systems (Applied Biosystems, Foster City, CA, USA).

For the quantitative analysis of the predicted target genes of miR-139-5p and miR-375, the expression levels were analyzed from cells transfected with respective miRNA precursors or negative control. RNA was converted into cDNA using the High-Capacity cDNA Reverse Transcription Kit (Applied Biosystems, Foster City, CA, USA). Real-time PCR analysis was performed with a 2× SYBR Select Master Mix (Applied Biosystems, Foster City, CA, USA) using ABI PRISM 7300 Real Time PCR System (Applied Biosystems, Foster City, CA, USA). *ACTB* was used as a reference gene. The primer sequences used in the study are listed in Table S4.

Relative miRNA and mRNA expression levels were compared between the studied groups by Wilcoxon test (eutopic vs. ectopic) or the Mann–Whitney U test (cells transfected with miRNA precursor vs. negative precursor control) and a *p*-value ≤ 0.05 was considered significant. The FC was calculated according to the 2^−ΔΔ*C*t^ method [47]. 

## Figures and Tables

**Table 1:** Most abundantly expressed miRNAs in endometrial eutopic and ectopic stromal cells.

miRNAs in Eutopic Stroma	Average Raw Read Count	miRNAs in Ectopic Stroma	Average Raw Read Count
let-7a-5p	262,062	let-7a-5p	336,278
miR-148a-3p	251,377	miR-10b-5p	185,323
let-7f-5p	171,896	miR-21-5p	149,525
miR-10b-5p	119,737	let-7f-5p	97,019
miR-21-5p	116,936	miR-148a-3p	89,056
miR-26a-5p	102,057	miR-99a-5p	88,792
miR-143-3p	98,860	miR-26a-5p	88,348
let-7i-5p	89,804	miR-143-3p	67,660
miR-99a-5p	89,793	let-7b-5p	51,762
miR-199a-3p	76,412	miR-126-3p	49,937

**Table 2:** Differentially-expressed miRNAs between ectopic and eutopic stromal cells identified by edgeR, DESeq2 and BaySeq programs.

miRNA ID	log_2_FC	FDR (edgeR)	padj (DESeq2)	FDR.DE (BaySeq)	Average CPM Eutopic Stroma	Average CPM Ectopic Stroma
**Upregulated miRNAs in ectopic stroma**						
**hsa-miR-139-5p**	5.0	1.4 × 10^−24^	7.2 × 10^−39^	1.5 × 10^−2^	57	1292
hsa-miR-139-3p	6.1	4.9 × 10^−24^	8.5 × 10^−29^	1.6 × 10^−2^	6	242
hsa-miR-202-5p	9.3	2.8 × 10^−19^	5.8 × 10^−11^	7.2 × 10^−3^	0	51
hsa-miR-506-3p	5.8	1.4 × 10^−17^	9.9 × 10^−17^	1.8 × 10^−2^	4	204
hsa-miR-150-5p	4.3	2.5 × 10^−14^	7.7 × 10^−17^	9.5 × 10^−3^	14	203
hsa-miR-202-3p	9.1	3.1 × 10^−14^	3.5 × 10^−9^	3.9 × 10^−2^	0	41
hsa-miR-150-3p	7.3	6.5 × 10^−12^	5.2 × 10^−6^	4.7 × 10^−3^	0	15
hsa-miR-513c-5p	5.6	1.1 × 10^−9^	2.2 × 10^−6^	1.9 × 10^−2^	1	19
hsa-miR-193a-5p	2.7	1.2 × 10^−9^	3.5 × 10^−14^	3.8 × 10^−2^	44	194
hsa-miR-584-5p	3.1	9.1 × 10^−7^	6.5 × 10^−5^	3.4 × 10^−2^	3	23
hsa-miR-371a-5p	4.5	1.1 × 10^−6^	7.2 × 10^−4^	2.9 × 10^−2^	1	11
hsa-miR-216b-5p	4.3	7.5 × 10^−5^	1.8 × 10^−3^	4.5 × 10^−2^	1	9
**Downregulated miRNAs in ectopic stroma**						
**hsa-miR-375**	−4.9	1.4 × 10^−14^	3.7 × 10^−11^	5.9 × 10^−3^	162	3
hsa-miR-105-5p	−4.7	1.6 × 10^−13^	4.4 × 10^−9^	2.1 × 10^−2^	104	3
hsa-miR-1298-5p	−5.8	2.5 × 10^−9^	5.6 × 10^−5^	1.3 × 10^−2^	18	0
hsa-miR-6507-5p	−4.8	5.2 × 10^−8^	3.6 × 10^−4^	3.6 × 10^−2^	27	1
hsa-miR-767-5p	−4.7	8.5 × 10^−8^	5.5 × 10^−4^	2.3 × 10^−2^	25	1
hsa-miR-675-3p	−3.3	1.9 × 10^−6^	7.7 × 10^−4^	3.1 × 10^−2^	29	2
hsa-miR-429	−4.4	1.9 × 10^−6^	2.3 × 10^−3^	4.1 × 10^−2^	23	1
hsa-miR-141-3p	−3.8	3.7 × 10^−5^	1.0 × 10^−2^	8.4 × 10^−3^	12	1
hsa-miR-873-5p	−3.5	3.9 × 10^−4^	4.6 × 10^−2^	4.3 × 10^−2^	9	1

miRNAs in **bold** were chosen for validation by qRT-PCR. FC—fold change, CPM—count per million, FDR—false discovery rate, DE—differentially expressed.

**Table 3:** Novel miRNAs detected from eutopic and ectopic stroma.

Provisional ID	Average Read Count in Eutopic Stroma	Average Read Count in Ectopic Stroma	Similarities with Other Human miRNAs	Consensus Mature Sequence	Precursor Coordinate, Forward (+) or Reverse (−) Strand
10_4598	1	2	-	gucauagacuagugcuuccga	10:106043903..106043987:−
12_4331	630	67	-	guucugggcuguagugagcuaugc	12:24706803..24706886:+
11_4914	9	11	-	aacugcucuucucuaauuuaa	11:101346555..101346607:−
19_4246	8	9	hsa-miR-25-3p	gugugugcaccugugucugucugu	19:18284682..18284741:+
3_22611	17	14	-	cucugggcugcagugcgcuaugc	3:49863521..49863597:−
3_18752	4	0	-	ugugguggcugcugcuggugc	3:53763045..53763105:+
6_24262	4	17	hsa-let-7b-5p	ugagguaguagguggugugc	6:158493843..158493925:−
8_30909	8	2	hsa-miR-9903	ccagccuacuggaggauaagagg	8:98393666..98393724:−

## Supplementary Materials

Supplementary materials can be found at http://www.mdpi.com/1422-0067/19/12/3789/s1.

## Abbreviations

CPM	count per million
FACS	fluorescence-activated cell sorting
FC	fold change
FDR	false discovery rate
miRNA	microRNA
qRT-PCR	quantitative real-time PCR
TF	transcription factor
