# Supplementary figures and images for: Differentially-Expressed miRNAs in Ectopic Stromal Cells Contribute to Endometriosis Development: The Plausible Role of miR-139-5p and miR-375
Source: Int J Mol Sci. 2018 Nov 28;19(12):3789. doi: 10.3390/ijms19123789 (PMC6321240; doi:10.3390/ijms19123789)

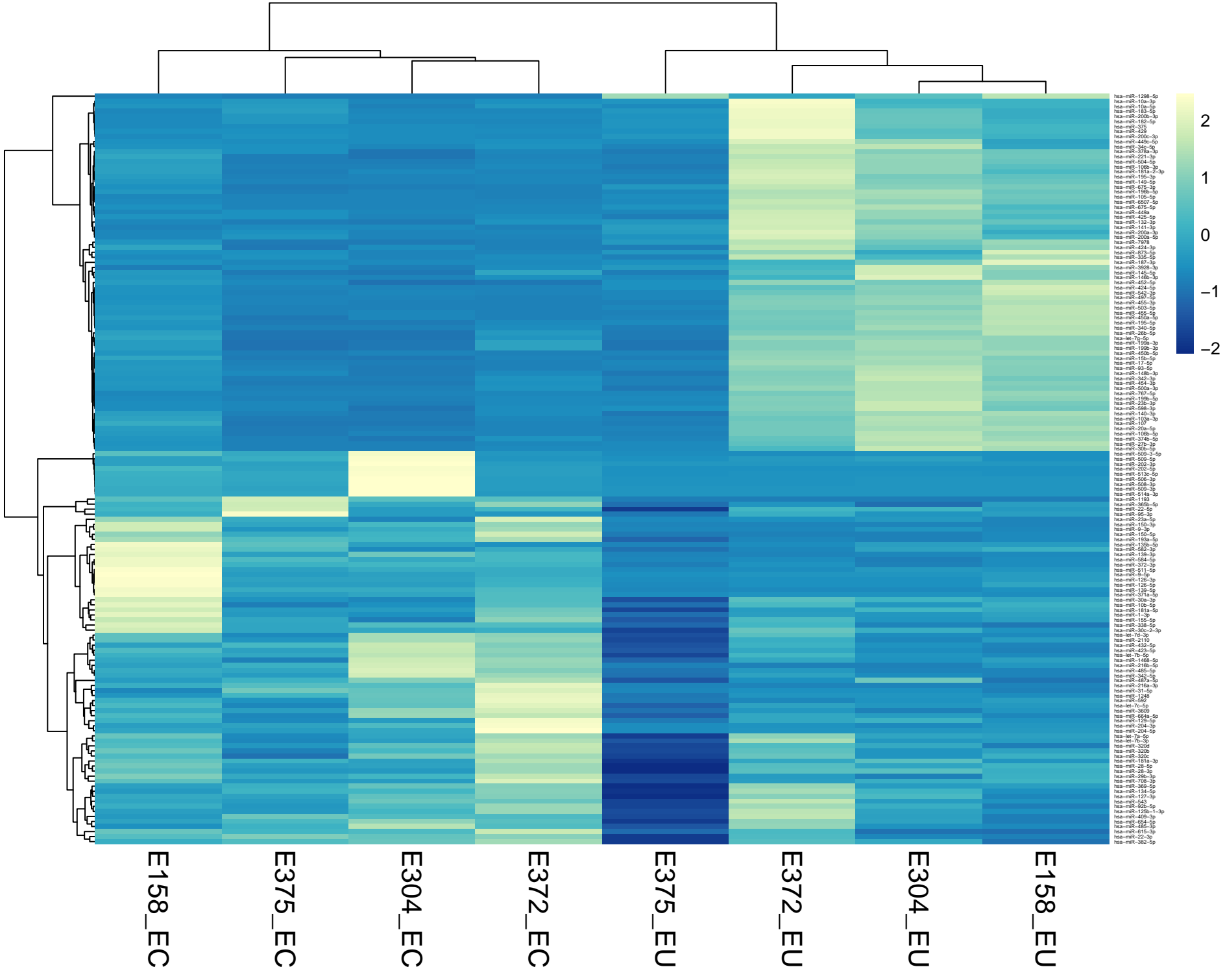

Supplement: Supplementary file 1 [file ijms-19-03789-s001.zip › Figure S1.pdf]

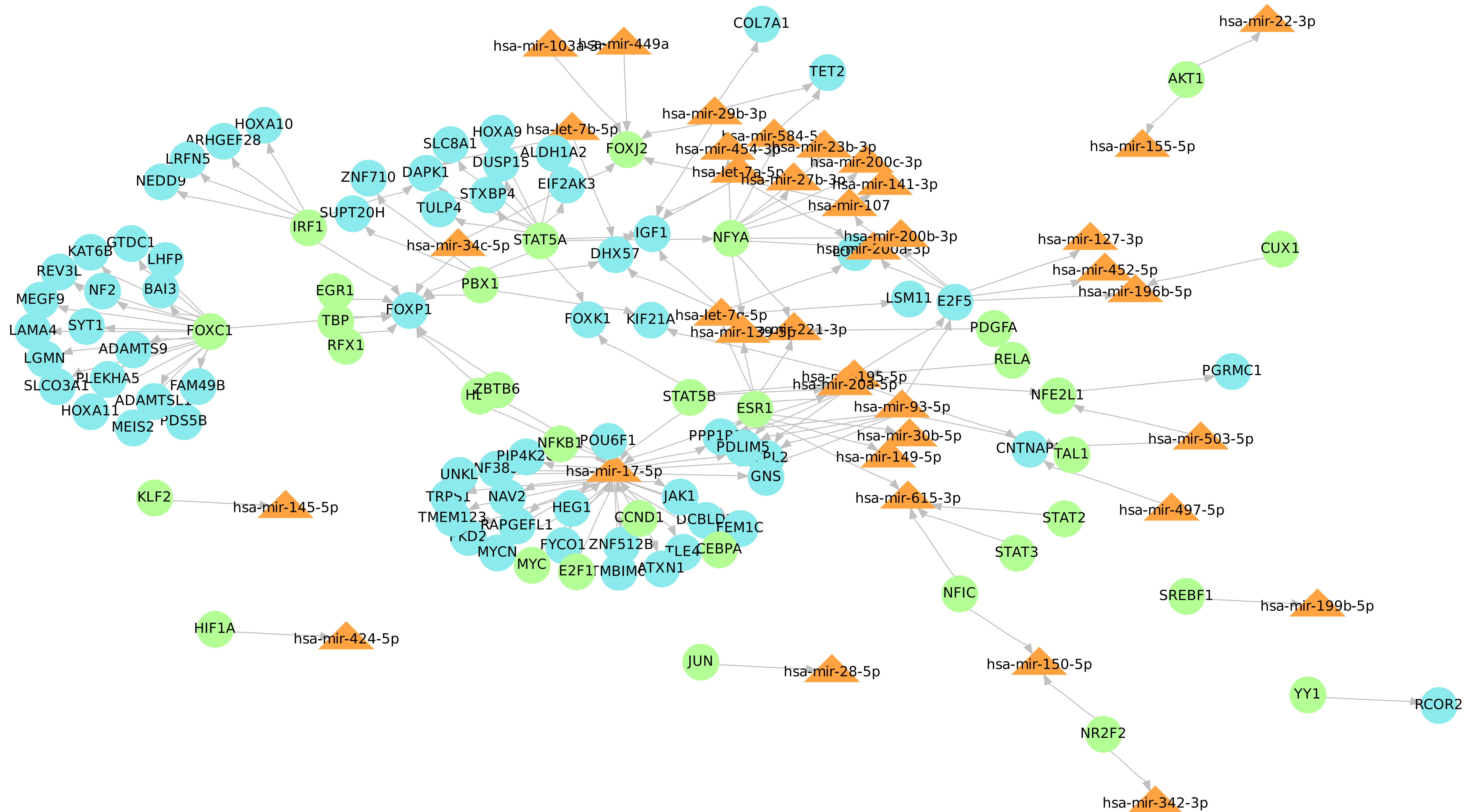

Supplement: Supplementary file 1 [file ijms-19-03789-s001.zip › Figure S2.pdf]
